# Identification of high-risk patients for referral through machine learning assisting the decision making to manage minor ailments in community pharmacies

**DOI:** 10.3389/fphar.2023.1105434

**Published:** 2023-07-11

**Authors:** Noelia Amador-Fernández, Shalom I. Benrimoj, Victoria García-Cárdenas, Miguel Ángel Gastelurrutia, Emma L. Graham, Rubén Palomo-Llinares, Julia Sánchez-Tormo, Vicente J. Baixauli Fernández, Elena Pérez Hoyos, Javier Plaza Zamora, Vicente Colomer Molina, Ricardo Fuertes González, Óscar García Agudo, Fernando Martínez-Martínez

**Affiliations:** ^1^ Pharmaceutical Care Research Group, University of Granada, Granada, Spain; ^2^ Center for Primary Care and Public Health (Unisanté), University of Lausanne, Lausanne, Switzerland; ^3^ Graduate School of Health, University of Technology Sydney, Sydney, NSW, Australia; ^4^ Department of Public Health and History of Science, University Hospital of Sant Joan d’Alacant, Alicante, Spain; ^5^ International Virtual Center for Nutrition Research (CIVIN), Alicante, Spain; ^6^ Spanish Society of Clinical, Family and Community Pharmacy, Madrid, Spain; ^7^ Pharmaceutical Association of Valencia, Valencia, Spain

**Keywords:** machine learning, community pharmacy services, triage, primary healthcare, general practice

## Abstract

**Background:** Data analysis techniques such as machine learning have been used for assisting in triage and the diagnosis of health problems. Nevertheless, it has not been used yet to assist community pharmacists with services such as the Minor Ailment Services These services have been implemented to reduce the burden of primary care consultations in general medical practitioners (GPs) and to allow a better utilization of community pharmacists’ skills. However, there is a need to refer high-risk patients to GPs.

**Aim:** To develop a predictive model for high-risk patients that need referral assisting community pharmacists’ triage through a minor ailment service.

**Method:** An ongoing pragmatic type 3 effectiveness-implementation hybrid study was undertaken at a national level in Spanish community pharmacies since October 2020. Pharmacists recruited patients presenting with minor ailments and followed them 10 days after the consultation. The main outcome measured was appropriate medical referral (in accordance with previously co-designed protocols). Nine machine learning models were tested (three statistical, three black box and three tree models) to assist pharmacists in the detection of high-risk individuals in need of referral.

**Results:** Over 14′000 patients were included in the study. Most patients were female (68.1%). With no previous treatment for the specific minor ailment (68.0%) presented. A percentage of patients had referral criteria (13.8%) however, not all of these patients were referred by the pharmacist to the GP (8.5%). The pharmacists were using their clinical expertise not to refer these patients. The primary prediction model was the radial support vector machine (RSVM) with an accuracy of 0.934 (CI95 = [0.926,0.942]), Cohen’s kappa of 0.630, recall equal to 0.975 and an area under the curve of 0.897. Twenty variables (out of 61 evaluated) were included in the model. radial support vector machine could predict 95.2% of the true negatives and 74.8% of the true positives. When evaluating the performance for the 25 patient’s profiles most frequent in the study, the model was considered appropriate for 56% of them.

**Conclusion:** A RSVM model was obtained to assist in the differentiation of patients that can be managed in community pharmacy from those who are at risk and should be evaluated by GPs. This tool potentially increases patients’ safety by increasing pharmacists’ ability to differentiate minor ailments from other medical conditions.

## Introduction

Community pharmacists’ scope of practice has been extending over time with the inclusion of services such as minor ailment services, medication therapy management or immunization are now under the pharmacists’ responsibility (Adams and Weaver, 2021; DiPietro Mager and Bright, 2022).

In Spain different pharmacy services are nationally defined and protocolized (ForoAF-FC, 2022), although not all of them are implemented or remunerated by the national health system. The minor ailment service (MAS) has been defined as the one “provided upon patient request in the pharmacy, when unsure of which medicinal product to acquire and upon requesting that the pharmacist provide the most appropriate remedy for a specific health problem” (ForoAF-FC, 2022).

In many other countries, such as the United Kingdom and Canada, MASs have been implemented to increase the capacity of primary care (Aly et al., 2018). Their main objectives are to reduce the burden of primary care consultations with general medical practitioners (GPs) allowing them to focus on more complex patients (Aly et al., 2018), to assist patients in choosing the appropriate level of care and to allow a better utilization of community pharmacist skills which extends their role in primary care (Butterworth et al., 2017; Bradley et al., 2018). A systematic review including studies from studies the United Kingdom, Europe, Australia, Canada, and Singapore, concluded that triage services in community pharmacy reduce the burden on other healthcare services by diverting those seeking treatment for minor ailments (Curley et al., 2016).

MAS includes clinical protocols for pharmacists to assist in the triage of patients that can be managed *in situ* or to those at risk that require referral to other healthcare professionals. One of the main characteristics of MASs is a series of clinical management pathways, which include referral criteria and red flags for minor ailments (Curley et al., 2016). Over sixty different minor ailments are included in protocols in international MASs in countries such as Scotland (gov.scot. NHS Pharmacy First Scotland: information for patients, 2020), England (Boots. NHS Minor, 2022), Wales (NHS. Common, 2019), Canada (CPhA, 2022) and Spain (Amador-Fernández et al., 2018). The protocols, that were mutually agreed with GPs, reflect the potential of community pharmacies to assess common problems that can frequently lead to consultations with GPs or emergency departments (Fielding et al., 2015).

MAS, with its underpinning clinical protocols, allows the differentiation of patients’ demands from patients’ needs in order to identify the most appropriate entry point into the healthcare system whilst ensuring patient safety (Robertson-Steel, 2006). There is evidence that pharmacist-led MASs improve healthcare outcomes (Amador-Fernández et al., 2019; Dineen-Griffin et al., 2021; Stämpfli et al., 2022).

More recently, technologies, such as machine learning, have been shown to be an important diagnostic tool assisting healthcare professionals (WHO, 2021). Machine learning is a field of artificial intelligence that focuses on the study of algorithms that learn from previous data (Mitchell, 1997). The World Health Organization has emphasized the future of artificial intelligence and its use in aiding decision-making by clinicians (WHO, 2021). Machine learning has already been used for aiding disease prognosis in cardiovascular diseases (Azmi et al., 2022), diagnosing neurodegenerative diseases (Myszczynska et al., 2020) and for classifying and predicting the progression of multiple sclerosis (Vázquez-Marrufo et al., 2022). The use of predictive models and their impact is said to be increasing exponentially (NCBI, 2016) and, although these models cannot replace GP diagnostic intelligence, it could augment it (Summerton and Cansdale, 2019).

Machine learning has been used in some pharmacy settings to detect adverse drug events (Bouzillé et al., 2019) and to evaluate patients’ adherence (Lo-Ciganic et al., 2015; Koesmahargyo et al., 2020) but it has not yet been used to assist community pharmacists provide triage services to appropriately identify high-risk individuals in need of referral. Therefore, there is a need of using advance technologies to assist pharmacists in their daily tasks of triaging patients consulting or demanding a treatment for a minor ailment in community pharmacy.

The aim of this study was to develop a predictive model for high-risk patients that need referral assisting community pharmacists’ triage through a MAS.

## Materials and methods

### Study design

Data for the development of the predictive model was derived from a pragmatic type 3 effectiveness-implementation hybrid study (Curran et al., 2012), which commenced in October 2020 and is ongoing, at a national level in Spanish community pharmacies. The study protocol was registered in ClinicalTrials.gov NCT05247333. A brief description of the study is as follows.

### Study population

Six Pharmaceutical Associations and the Spanish Society of Clinical, Family and Community Pharmacy (SEFAC) invited their member community pharmacies/pharmacists to take part in the program through a number of channels. Patients included in the study were those who presented in the participating community pharmacies with symptoms or requested a medication for a minor ailment. The patient inclusion criteria were: aged ≥18 years, or younger if they were accompanied by a responsible adult, presenting one of the minor ailments listed below or other included at the discretion of the pharmacist. Clinical protocols were designed for the management of: upper respiratory tract related (nasal congestion, cold, cough); pain related (headache, joint and back pain, dental pain, sore throat, dysmenorrhea); gastroenterology (heartburn, flatulence, diarrhea, constipation, vomiting); dermatological (acne, mouth ulcers, dermatitis, soft tissue injuries, cold sore hyperhidrosis, bites and stings, athlete’s foot, burns, rashes); other ailments (acute stress disorder, fever, hemorrhoids, insomnia, red eye, dry eye, vaginal candidiasis, varicose veins). These protocols were agreed through a co-design process between medical and pharmacy associations and were facilitated by researchers from the University of Granada.

### Description of the intervention

The pharmacist-patient intervention is described using the Template for Intervention Description and Replication or TIDieR (de Barra et al., 2019) template (Supplementary Appendix SA1). It included a standardized consultation protocol incorporating:

• A general procedure for the service (Pharmaceutical Care Forum in Community Pharmacy FA-F, 2019).
• Management protocols co-developed between GPs and community pharmacists for each specific minor ailment, including referral criteria, and recommended pharmacological and non-pharmacological treatment (Amador-Fernández et al., 2018).
• A web-based software program (SEFAC eXPERT^®^) (SEFAC. SEFAC, 2014) that guided pharmacists

Educational training was provided covering topics such as service provision, service protocols, communication skills with the patient and other health professionals, web-based software use and data collection. Change agents (CAs) were used to conduct the training as well as periodic follow-ups in order to provide feedback and advice to the pharmacists as well as checking the fidelity to the intervention. CAs also received monthly ongoing training, implementation strategies (Damschroder et al., 2009) and feedback by the research group.

### Study outcomes

The outcome variables measured related to the impact of MAS were (Amador-Fernández et al., 2022):

• Appropriate medical referral: patient referral by the pharmacist made in accordance with the co-designed protocols.
• Modification of direct product request.
• Symptom resolution: relief of symptoms from 1“not at all” to 10“completely” at 10-day follow-up.
• Re-consultation rate for the same minor ailment 10 days or after the pharmacist consultation.

This paper will report only on the medical referral model.

### Ethics approval and consent to participate

The study was approved by the University of Granada Ethics Committee (registration number 253c23c678ee33a14d3dc16e585baf6a5a3da056). Participating pharmacists had to sign a commitment form to participate in the study. Patients or responsible adults were requested to provide written consent after being informed of the study.

### Machine learning model

Data used for obtaining the model was extracted from the web-based consultation program. For the model development, the outcome was appropriate medical referral defined as a dichotomous qualitative variable (yes/no).

There were thirteen patient-related covariates: gender, age, province, other health problems, other treatments, minor ailment consulted, previous treatment of the minor ailment, minor ailment duration, referral criteria (due to the patient’s age, pregnancy/breastfeeding, red flags, symptom duration, other treatments, health problems and others), consultation type (presenting in CP with a consultation/requesting a medication for a minor ailment), quality of life (visual analogue scale from 0 to 100), physiological status (pregnancy/breastfeeding) and day of the consultation.

Pharmacists-related covariates included: gender, age, nationality and role in the CP. Pharmacies-related covariates were location (rural/urban), location size and number of pharmacists working in the CP.

### Statistical analysis and design of the models

Quantitative covariates were described using the arithmetic mean, standard deviation, median, maximum, and minimum as appropriate. Near zero variance predictors were removed. Furthermore, two non-relevant variables with more than 65% of missing values were eliminated (Anatomical Therapeutic Chemical or ATC classification of the recommended treatments and whether the treatment requested was a prescription treatment) (Kang, 2013). Remaining variables were subjected to missing value imputation using k-Nearest Neighbors. Outliers were identified and treated using the Tukey’s boxplot method for the covariate symptom duration and Winsorization for existing underlying health problems and the number of treatments being used (Aguinis et al., 2013). In addition, a Yeo-Johnson transformation was used to stabilize variance for the three covariates (symptom duration, underlying health problems and the number of treatments being used) and additionally for EQVAS and number of pharmacists per community pharmacy (Yeo and Johnson, 2000). Finally, correlation between covariates was analyzed to avoid multicollinearity problems. 70.0% of the database was used for obtaining the models as a training cohort and 30.0% as a test cohort (Lantz, 2019).

For the main prediction model, an additive selection technique with three feature selection methods: Kohonen’s Learning Vector Quantization (LVQ) algorithm (Kohonen, 1995), Random Forest and RFE (recursive feature elimination) was used to select the number of predictors that should be included in the model (Kuhn and Johnson, 2013; Kuhn and Johnson, 2019).

Due to the high-class imbalance in the referral outcome variable (no: 91.5%, yes: 8.5%), the SMOTE (Synthetic Minority Over-sampling Technique) was applied to the training dataset to reduce the class imbalance (Chawla et al., 2002). Nine models were tested for the outcome “referral to the GP”: three statistical models (k-Nearest Neighbor or kNN, Naive Bayes or NB, Logistic Regression or RegLog), three black box models (Artificial Neural Network or ANN, Support Vector Machine Lineal or SVM. L, Support Vector Machine Radial or SVM. R) and three tree models (C5.0 Decision Tree or C5DT, Random Forest or RF, XGBoost or XGB).

To assess the primary prediction model, its performance was evaluated for the 25 most frequent patient profiles and for the 25 most frequent pharmacists’ profiles found in the study. Accuracy, Cohen’s kappa and recall were evaluated for each profile.

The software used for the analysis was R v4.2.2 with the package RStudio 2022.12.0 build 353.

## Results

14,083 patients were included in the study with patient characteristics presented in Table 1. Most participant patients were female (68.1%, *n* = 9594), with a consultation about the minor ailment (84.6%, *n* = 11911), with no previous treatment for the minor ailment consulted (68.0%, *n* = 9588) and with no referral criteria (86.2%, *n* = 12146).

**TABLE 1:** Patients’ characteristics.

	Number (n)	Proportion (%)
	(N = 14,083)	
Gender		
Female	9594	68.1
Male	4486	31.9
Minor ailment consulted		
Dermatological	2603	18.5
Gastroenterology	2199	15.6
Pain	2752	19.5
Respiratory	1814	12.9
Other	4715	33.5
Consultation type		
Consultation	11911	84.6
Direct product request	2172	15.4
Previous treatment of the minor ailment		
Yes	4495	31.9
No	9588	68.0
Patients with referral criteria		
Yes	1937	13.8
No	12146	86.2
Reason for referral^a^		
Red flags	846	39.5
Patient’s age	398	18.6
Symptom duration	361	16.9
Treatments	263	12.3
Health problems	186	8.7
Pregnancy/Breastfeeding	38	1.8
Other	47	2.2
Physiological status		
Breastfeeding	127	0.9
Pregnancy	0	0.0
None	13956	99.1

^a^ A patient could have more than one referral criteria.

Most patients did not present any referral criteria (86.2%, *n* = 12,116). Those with referral criteria presented a mean of 1.02 (SD = 0.56) criteria with a maximum of three criteria. Red flags were the most common referral criteria (39.5%, *n* = 846) (Table 1). Higher number of patients presented referral criteria (13.8%, *n* = 1937) than those who were ultimately referred (8.5%, *n* = 1,194). A synthetic minority oversampling technique (SMOTE) was used to solve the class imbalance.

For obtaining the machine learning model, 70.0% (*n* = 9,839) of the data set was used as a training cohort and 30.0% (*n* = 4,218) as a test cohort. Correlation between covariates was analyzed without finding any high correlations. A moderate correlation was found between the patients’ health problems and their medication (0.29); nevertheless, no additional correction was made due to the correlation being lower than 0.5.

**FIGURE 1:**
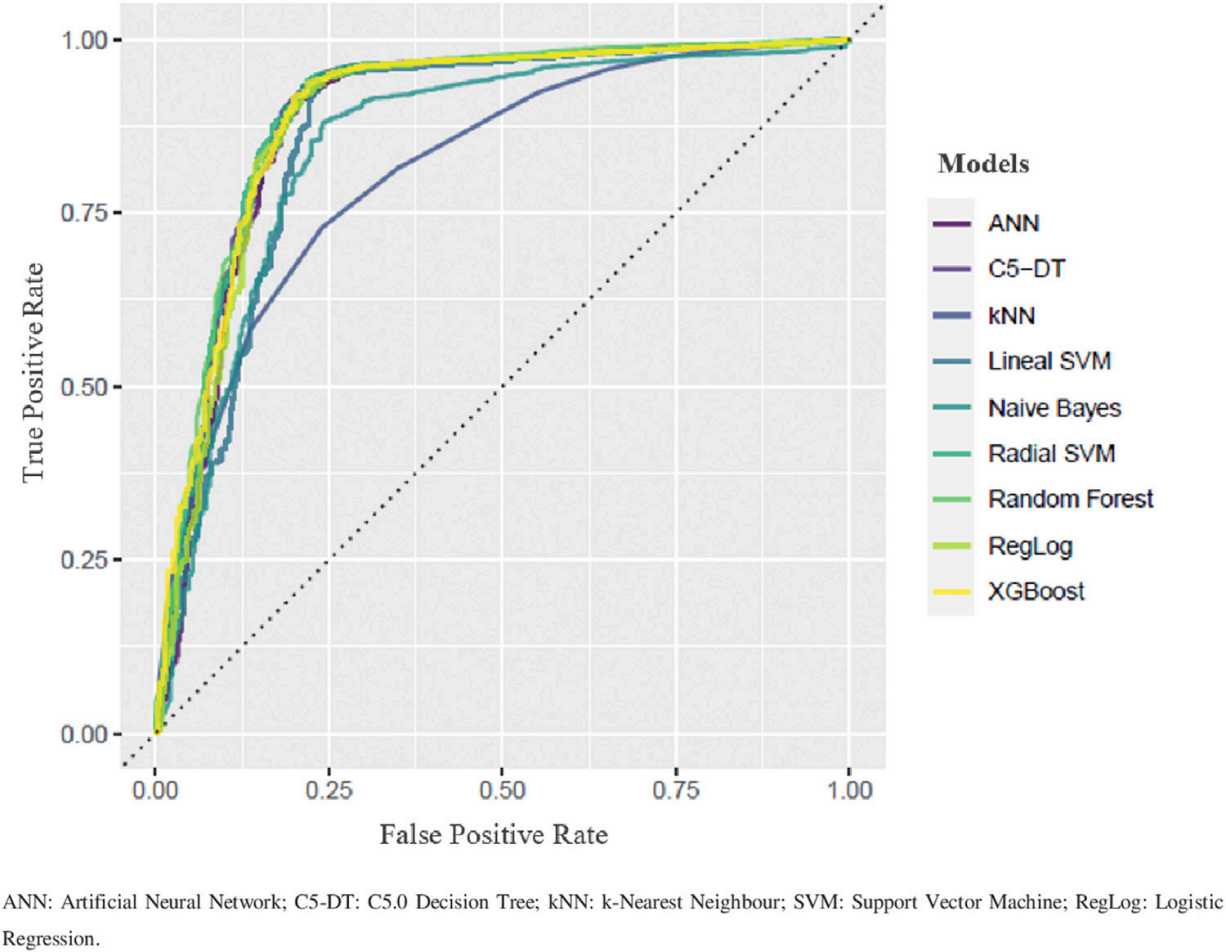
Comparative Roc curves.

**FIGURE 2:**
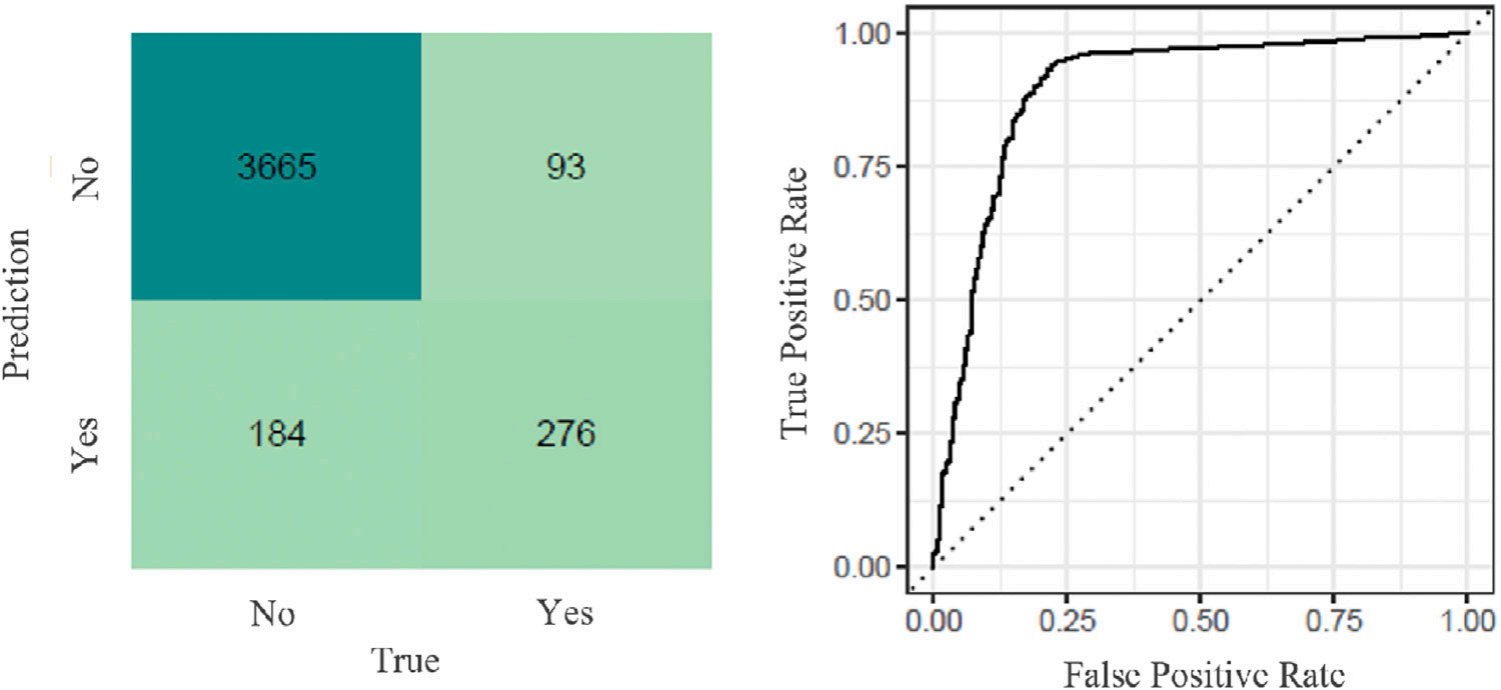
Radial Support Vector Mchine Model:confusion matrix and Roc curve.

The primary prediction model (Figure 1) was the radial support vector machine (RSVM) with an accuracy of 0.934 (CI95 = [0.926,0.942]), Cohen’s kappa of 0.630, recall equal to 0.975 and an area under the curve of 0.897 (Figure 2).

The confusion matrix for the primary prediction model (RSVM) (Figure 2) shows that the model could predict 95.2% (*n* = 3665) of the true negatives (patients that would not be referred to the GP according to the model and should not be referred) and 74.8% (*n* = 276) of the true positives (patients that would be referred to the GP according to the model and should be referred).

**FIGURE 3:**
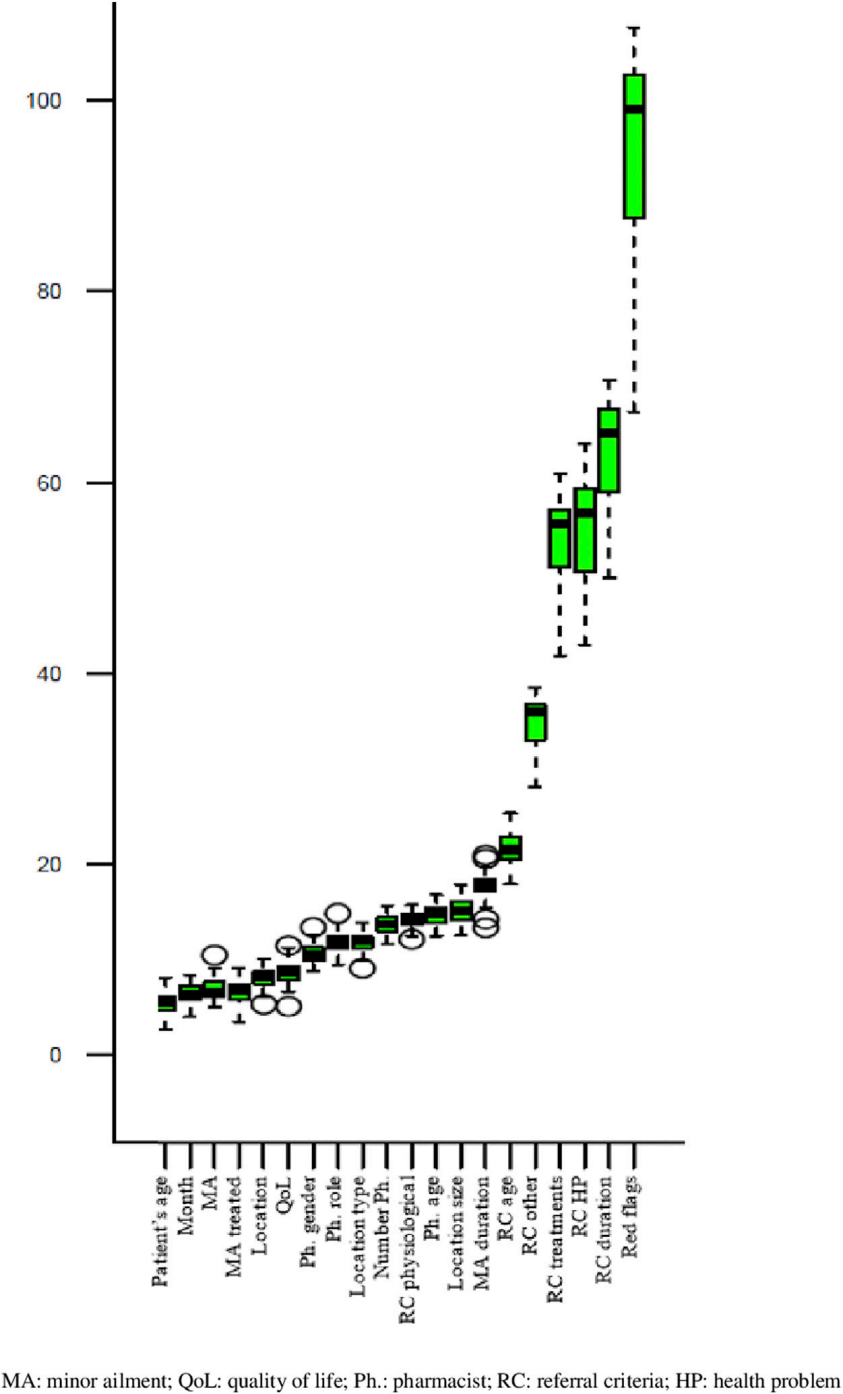
Feature importance of the Variable (Boruta algorithems).

Twenty variables (out of 61 covariates evaluated) were finally included in the model, i.e., the variables that showed statistically significant difference (*p* ≤ 0.01) when tested compared to a random variable using a random forest model. These variables were listed according to relevance (Figure 3): referral criteria (red flags, symptom duration, health problems, treatments, patients’ age), symptom duration, CP population served, pharmacist’s age, physiological status, number of pharmacists working in the CP, CP’s location, pharmacist’s role in the CP, pharmacist’s gender, quality of life, previous treatment of the minor ailment, minor ailment consulted, month of the consultation and patient’s age.

The first six covariates included in the primary prediction model were related to the patient presenting with criteria that might lead to referral (Figure 3). The importance of these referral criteria was compared to a random covariate. Red flags increase the odds of experiencing referral by 94.4% (IC95 = [91.8, 96.9]), symptom duration by 63.0% (IC95 = [61.7, 64.4]), other health problems unrelated to the minor ailment consulted by 55.4% (IC95 = [54.1, 56.6]), treatments to treat other health problems by 54.0% (IC95 = [53.0, 55.1]), other criteria by 34.9% (IC95 = [34.2, 35.5]) and patient’s age by 21.8% (IC95 = [21.4, 22.2]).

The 25 most prevalent patients’ profiles were extracted to evaluate performance of the model (Table 2). The model was considered appropriate (accuracy, Cohen’s kappa and recall over 0.62) for 56% (14/25) of the profiles. In addition, the 25 most prevalent pharmacists’ profiles were extracted (Table 3) and the model identified eight lower performance profiles (Cohen’s kappa <0.62) when triaging patients.

**TABLE 2:** Accuracy of the Radial Support Vector Machine model for 25 patients’ profiles included in the study.

Patients’ profile	NO. Patients study (N = 14083)	NO. Patients test cohort (N = 4,218)	Gender	Age	Minor ailment group	Minor ailment	Symptom duration (days)	Accuracy	Cohen’s kappa	Recall
1	173	56	Female	Older	Pain	Joint pain	2–5	0.946	0.638	0.962
2	133	41	Female	Older	Gastroenterology	Constipation	2–5	0.927	0.685	0.944
3	132	41	Female	Adult	Other	Vaginitis	2–5	0.902	0.547	0.971
4	132	35	Female	Older	Pain	Joint pain	1	0.971	0.653	1
5	119	29	Female	Adult	Pain	Joint pain	2–5	0.966	<0.450	0.966
6	109	36	Female	Older	Other	Other	1	0.889	0.600	0.933
7	100	27	Female	Adult	Other	Other	1	0.963	<0.450	1
8	95	29	Female	Adult	Respiratory	Cold	2–5	0.931	<0.450	0.964
9	90	19	Female	Older	Dermatological	Dermatitis	2–5	1	1	1
10	85	29	Female	Adult	Pain	Joint pain	1	0.931	0.628	0.962
11	84	31	Female	Older	Other	Other	2–5	1	1	1
12	82	28	Male	Adult	Pain	Joint pain	1	0.929	0.632	0.923
13	80	30	Male	Adult	Pain	Joint pain	2–5	1	1	1
14	79	23	Female	Adult	Gastroenterology	Constipation	2–5	1	1	1
15	79	32	Female	Adult	Respiratory	Cough	2–5	0.969	0.784	0.967
16	78	29	Female	Adult	Other	Other	2–5	0.931	<0.450	0.964
17	78	22	Female	Older	Respiratory	Cough	2–5	0.955	<0.450	1
18	76	27	Female	Adult	Respiratory	Nasal congestion	2–5	0.963	<0.450	0963
19	76	20	Female	Adult	Dermatological	Dermatitis	2–5	0.900	<0.450	0.947
20	74	15	Male	Older	Pain	Joint pain	2–5	0.867	<0.450	0.867
21	74	25	Female	Older	Pain	Joint pain	6–10	0.880	<0.450	0.880
22	71	20	Female	Older	Other	Other	6–10	0.950	0.773	1
23	69	17	Male	Older	Gastroenterology	Constipation	2–5	1	1	1
24	69	16	Female	Young	Other	Vaginitis	2–5	1	1	1
25	68	22	Female	Adult	Other	Vaginitis	1	1	1	1

**TABLE 3:** Accuracy of the Radial Support Vector Machine model for 25 pharmacists’ profiles included in the study.

Patients’ profile	NO. Patients study (N = 14083)	NO. Patients test cohort (N = 4,218)	Gender	Age	Minor ailment group	Minor ailment	Symptom duration (days)	Accuracy	Cohen’s kappa	Recall	Patients’ profile
1	490	150	Owner	Female	55–64	Capital	>500	2–4	0.967	0.744	0.979
2	313	96	Employee	Female	35–44	Town	5–10	>5	0.990	0.795	1
3	277	85	Employee	Female	45–54	Capital	>500	2–4	0.929	0.744	0.932
4	264	81	Employee	Female	35–44	Capital	100–500	2–4	0.914	<0.450	0.934
5	245	73	Employee	Female	35–44	Town	50–100	2–4	0.932	0.629	0.955
6	183	60	Owner	Male	55–64	Capital	>500	2–4	0.950	0.640	0.964
7	166	45	Owner	Female	45–54	Capital	>500	2–4	0.978	0.845	0.976
8	162	47	Owner	Female	55–64	Town	5–10	>5	0.936	<0.450	0.957
9	160	48	Employee	Male	35–44	Town	50–100	2–4	1	1	1
10	160	46	Employee	Female	35–44	Town	100–500	2–4	1	1	1
11	157	37	Employee	Male	35–44	Capital	100–500	2–4	0.946	0.637	0.971
12	145	46	Employee	Female	<35	Capital	>500	2–4	0.978	0.911	0.975
13	137	34	Employee	Female	<35	Town	10–20	>5	0.941	0.719	1
14	135	39	Employee	Female	<35	Village	5–10	2–4	0.949	0.723	0.944
15	128	44	Employee	Female	55–64	Capital	100–500	2–4	0.977	0.788	1
16	123	36	Owner	Female	45–54	Town	10–20	2–4	0.944	0.471	0.971
17	117	43	Owner	Female	45–54	Town	30–50	2–4	0.953	0.645	1
18	114	31	Owner	Female	45–54	Village	100–500	1	1	1	1
19	112	25	Employee	Male	<35	Town	10–20	>5	0.920	0.457	0.957
20	112	23	Employee	Female	45–54	Village	10–20	2–4	0.696	<0.450	0.714
21	111	35	Employee	Male	35–44	Town	5–10	>5	0.971	<0.450	0.971
22	111	42	Owner	Female	45–54	Capital	100–500	2–4	0.929	0.690	0.921
23	109	33	Employee	Male	45–54	Capital	>500	2–4	0.788	0.512	0.826
24	105	37	Employee	Female	45–54	Town	100–500	2–4	0.892	0.537	0.938
25	105	36	Owner	Female	55–64	Town	30–50	2–4	1	1	1

## Discussion

Machine learning technologies are increasingly being developed and used as a diagnostic tool assisting healthcare professionals. Nevertheless, its use is not extended in community pharmacy, and it has never been developed for assisting pharmacists while triaging patients with minor ailments. This study developed a machine learning model (RSVM) to assist community pharmacists when triaging patients to better identify high-risk patients in order to differentiate those that may be managed by a community pharmacist from those who should be referred and evaluated by a GP. The model highlighted the most important patient characteristics that could predict which patients have a high probability of referral (prediction of 95.2% of the true negatives and 74.8% of the true positives). It also allowed the detection of certain pharmacists’ profiles that would benefit from supplementary training.

No similar studies have been carried out in community pharmacy. Nevertheless, studies in healthcare have used machine learning techniques in other settings to assist diagnosis. A systematic review that compared machine learning techniques for cardiovascular disease prediction found models with an accuracy between 81% and 100% concluding that the random forest model produced the most significant outcomes in terms of accuracy (Azmi et al., 2022). Our RSVM model obtained better results in terms of accuracy, area under the curve and recall than those studies for the prediction of medication adherence (Koesmahargyo et al., 2020). The model included 20 variables that showed statistically significant difference when tested, similarly to those included in a model used to predict asthma exacerbation in an upcoming year (Jiao et al., 2022).

## Implications for research and practice

Although, studies have reported that patients can be appropriately triaged in community pharmacy to GPs (Dineen-Griffin et al., 2020; Amador-Fernández et al., 2022); the use of predictive modelling at the time of the consultation would suggest, although not proven, a contribution to the increase in patient safety, clinical decision-making and governance. Future research should focus on developing artificial intelligence techniques to help clinicians in daily practice, always taking into consideration the legal and ethical aspects.

There are two main implications for practice: an increase in patients’ safety and improved community pharmacy practice. Interestingly, a study found that pharmacist self-perception to differentiate minor ailments from similar conditions has been found to have a low score when evaluating competencies to manage minor ailments such as recommendation of appropriate over the counter medicines (Makhlouf et al., 2021). Clinical protocols and guidelines together with machine learning models may increase pharmacists’ ability to differentiate minor ailments from other medical conditions. Secondly, a key element when triaging and/or diagnosing is not only the patients’ characteristics but also the health providers’ traits and their abilities to perform clinically. The model detected certain pharmacists’ profiles on which the CAs could focus their interventions, intensify training and perform more frequent follow-ups with the ultimate aim of improving patients’ clinical results.

## Strengths and limitations

To our knowledge, this is the first study using machine learning to develop a predictive model that can assist community pharmacists delivering MASs to triage patients. A major strength is that all data used to construct the model emanated from data derived from actual practices. New data will be used for the continuing learning of the model, permitting further improvement of the predictive ability of the model (now it was considered appropriate for 56% of the 25 most common patients’ profiles included in the study).

One of the main limitations of the study was the lack of availability of individual patient clinical and medical history, as community pharmacies do not have access to patient clinical history. The model was therefore restricted to the data obtained during the pharmacist-patient consultation, reflecting usual practice. Model performance could potentially be improved by adding more specific biomarkers that allow patient differentiation, which currently is not feasible in real practice for community pharmacy.

## Data Availability

The raw data supporting the conclusions of this article will be made available by the authors, without undue reservation. Requests to access these datasets should be directed to noelia.af@outlook.com.
