# Supplementary material for: Identification of high-risk patients for referral through machine learning assisting the decision making to manage minor ailments in community pharmacies
Source: Front Pharmacol. 2023 Jul 11;14:1105434. doi: 10.3389/fphar.2023.1105434 (PMC10368471; doi:10.3389/fphar.2023.1105434)
Supplement: Supplementary file 1 [file DataSheet1.PDF]

S1 Appendix

TIDieR
Template for Intervention Description and Replication

The TIDieR (Template for Intervention Description and Replication) Checklist*:

Information to include when describing an intervention and the location of the information

Number	Item	Where located **
1.	BRIEF NAME	Minor ailment service (MAS).
2.	WHY	MAS is defined as a "professional service provided upon patient's request in the pharmacy when unsure of which medicinal product to acquire […] for a specific health problem" (Pharmaceutical Care Forum in Community Pharmacy, 2019). The international literature reports that structured minor ailments schemes improve the clinical, humanistic and economic outcomes for patients and the health care system (1-4). Collaboratively agreed protocols have been shown to enhance appropriate referrals by pharmacist to other health care professionals, particularly to general medical practitioner. Appropriate referrals increase the quality of the service and increase patient's safety. Monitoring patient self-selection of non-prescription medication enhances quality use of these medicines and increases patient safety.<br>At present there appears to be wide variability of practices on how pharmacist respond to patients presenting symptoms and to self-medication product request. Quality standards need to be applied to promote the safe and effective management of minor ailments in community pharmacy setting.
3.	WHAT	The intervention was composed of:<br>Standardised consultation on an IT platform for pharmacist–patient intervention which included:<br>1. A MAS was provided following good pharmacy standards by the Pharmaceutical Care Forum in Community Pharmacy (see Procedures section).<br>2. Each minor ailment studied had a collaboratively agreed protocol (see Procedures section).

		3. Educational material for the patient included non-pharmacological treatment for each minor ailment: https://www.sefac.org/system/files/2020-01/INDICA%2BPRO_Informe.pdf (Appendix 4, pages 151 to 157)<br>4. An IT practice program (SEFAC eXPERT®) led the pharmacists through the individual patient consultation with selected pop ups including protocol flow and referral criteria for each minor ailment. It guided pharmacists through two main pathways: patients presenting with symptoms or requesting a non-prescription medicine. An example can be found on: https://www.sefac.org/system/files/2020-01/INDICA%2BPRO_Informe.pdf (Appendix 7, pages 160 to 163).<br>A change agent (CA) made regular on-site visits during the study to resolve doubts, problems, advice, educate pharmacists and check fidelity of the intervention through data inspection. Support was also provided via email/telephone during the study period. CA was trained to ensure the study objectives were met.<br>Educational training for pharmacists: Three half-day course (12 hours) was delivered by two experts (a community pharmacist and a general medical practitioner) which included: MAS procedure, good practice standards, agreed service protocols, communication's skills with the patient and other health professionals and data collection methods.
**4.**	**Procedures**	Pharmaceutical Care Forum in Community Pharmacy in 2010 has standardized procedures for the delivery of a MAS in community pharmacy. These were adapted and enhanced in the IT consultation process https://www.farmaceuticos.com/wp-content/uploads/2021/02/ON_GUIA_SPFA_FORO_2022_ING_PGs.pdf (Page 28 to 35)<br>https://www.farmaceuticos.com/wp-content/uploads/2019/09/BBPP-02-ENG-Servicio-Indicacion-Medicamentos.pdf

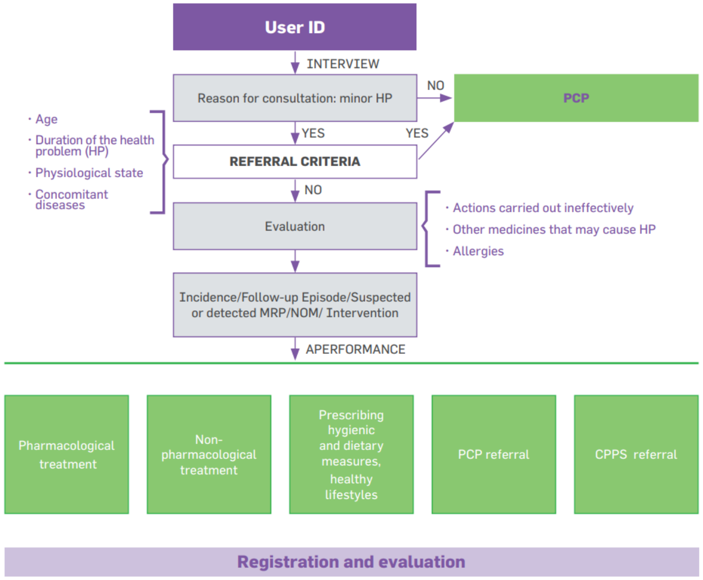

Figure 1. Diagram of the Minor Ailment Service procedure

*PCP: Primary Care physician; MRP: medication related problem; NOM: negative outcome associated with the medication; CPPS: clinical professional pharmacy service

Pharmaceutical Care Forum in Community Pharmacy developed this standard operational procedure (SOP) and established the requirements for providing MASs in the document "Good pharmacy practice in Spanish community pharmacy".

Specific protocols for each symptom are published in "Protocols for the MAS and Referral Criteria for Minor Ailments" (5). These protocols and guideline were codesigned and agreed between community pharmacists and general medical practitioners and include referral criteria according to patient's age, symptoms' duration, red flags, other health problems and special physiological situations such as pregnancy, breastfeeding; appropriate pharmacological and non-pharmacological treatment for each specific minor ailment. An example for one of the minor ailment's protocol (nasal congestion) can be found in Appendix 1 of: Amador-Fernández N, Benrimoj SI, Baixauli VJ, Climent MT, Colomer V, Esteban O et al. Colaboración farmacéutico-médico en la elaboración de protocolos consensuados para el tratamiento

		de síntomas menores: programa 'INDICA+PRO'. Farmacéuticos Comunitarios. 2019;11:21-31. https://doi: 10.5672/FC.2173-9218.(2019/Vol11).004.03.(6)
**5.**	**WHO PROVIDED**	Registered pharmacists provided the MAS after attending a 12-hour training and following the advice of a CA.<br>Pharmacy staff other than the pharmacists were not included in the study.
**6.**	**HOW**	MAS was provided through a face-to-face encounter between the pharmacist and the patient, so individual interviews were carried out in the community pharmacy. When patients attended the pharmacy either requesting a direct product request (non-prescription medicine) of presenting symptoms covered in the study they were informed about the study. If they accepted to participate the signed a consent form.<br>10 days following this consultation a researched phoned them at the number provided.
**7.**	**WHERE**	National level in Spain, community pharmacies were invited by six Pharmaceutical Associations and the Spanish Society of Clinical, Family and Community Pharmacy (SEFAC) to take part voluntarily in the program through a number of channels (email, websites, newsletters, advertisements).
**8.**	**WHEN and HOW MUCH**	The intervention was provided by the community pharmacist in a single consultation that took place when the patient visited the pharmacy asking for advice or requesting a non-prescription medication for one of the minor ailments included (see Tailoring).<br>The consultation using an IT practice program took a mean time of 5.48 minutes.
**9.**	**TAILORING**	Patients included in the study were those who presented in the participating community pharmacies with symptoms or requested a medication for a minor ailment. The patient inclusion criteria were: aged ≥18 years, or younger if they were accompanied by a responsible adult, presenting one of the minor ailments listed below or other included at the discretion of the pharmacist. Clinical protocols were designed for the management of: upper respiratory tract related (nasal congestion, cold, cough); pain related (headache, joint and back pain, dental pain, sore throat, dysmenorrhea); digestive (heartburn, flatulence, diarrhoea, constipation, vomiting); dermatological (acne, mouth ulcers, dermatitis, soft tissue injuries, cold sore hyperhidrosis, bites and stings, athlete's foot, burns, rashes); other ailments (acute stress disorder,

		fever, haemorrhoids, insomnia, red eye, dry eye, vaginal candidiasis, varicose veins). These protocols were agreed through a co-design process between medical and pharmacy associations, and were facilitated by researchers from the University of Granada. They included referral criteria and treatment to be evaluated depending on patient's characteristics.<br>Standardised consultation could follow two main pathways depending on patients presenting with symptoms or requesting a medicine for a minor ailment. Those cases where the patient requested a medicine, the pharmacists had to evaluate if the medication requested was the most appropriate treatment.
**10.** ǂ	**MODIFICATIONS**	No changes were made in the intervention during the course of the study.
**11.**	**HOW WELL (planned)**	The MAS scheme was co-designed with pharmacist, general medical practitioners, patients' organisations and local health administrators using existing materials and nationally agreed documentation. The specific pharmacist/patient interventions were developed with this group over a six-month period and then piloted.<br>A CA was trained to follow up the intervention and control pharmacists. He/she made regular visits/calls in every community pharmacy to assess adherence to the guidelines. The CA completed a checklist at each pharmacy including the collection of facilitators and barriers for delivering the service. The CA check the fidelity of the intervention through checks on the IT program and the extracted data from this program. The CA was available telephone and email contacts to assist pharmacists in the intervention group throughout the study.
**12.**ǂ	**HOW WELL (actual)**	In addition to further assess intervention adherence and fidelity, IT data collection program was checked by the research group. The consultation was documented though the IT practice program. The pharmacist recorded his/her actions in the program. Evidence for those cases where the community pharmacists didn´t adhere to the guidelines, for example, when referral criteria was detected by the pharmacists but the patient was not appropriately refer and those cases where the pharmacists recommended modification of the treatment requested but the patient did not follow the recommendation were recorded as part of the process.

1. Amador-Fernández N, Benrimoj S, Olry de Labry Lima A, García-Cárdenas V, Gastelurrutia M, Berger J, et al. Strengthening patients' triage in community pharmacies: a cluster randomised controlled trial to evaluate the clinical impact of a minor ailment service PloS one. 2022.

2. Dineen-Griffin S, Benrimoj SI, Rogers K, Williams KA, Garcia-Cardenas V. Cluster randomised controlled trial evaluating the clinical and humanistic impact of a pharmacist-led minor ailment service. BMJ quality & safety. 2020;29(11):921-31.
3. Paudyal V, Watson MC, Sach T, Porteous T, Bond CM, Wright DJ, et al. Are pharmacy-based minor ailment schemes a substitute for other service providers? A systematic review. The British journal of general practice : the journal of the Royal College of General Practitioners. 2013;63(612):e472-81.
4. Watson MC, Ferguson J, Barton GR, Maskrey V, Blyth A, Paudyal V, et al. A cohort study of influences, health outcomes and costs of patients' health-seeking behaviour for minor ailments from primary and emergency care settings. BMJ open. 2015;5(2):e006261.
5. Amador-Fernández N, Amariles P, Baixauli-Fernández V, Benrimoj S, Climent-Catalá M, Colomer-Molina V, et al. Protocolos de Indicación Farmacéutica y Criterios de Derivación al Médico en Síntomas Menores [Protocols for the minor ailment service and referral criteria for minor ailments]. Granada: Editorial Técnica Avicam; 2018.
6. Amador-Fernández N, Benrimoj S, Baixauli V, Climent M, Colomer V, Esteban O, et al. Colaboración farmacéutico-médico en la elaboración de protocolos consensuados para el tratamiento de síntomas menores: programa ‘INDICA+PRO’ [Colaboration between pharmacists and general medical practitioners for the elaboration of agreed protocols for minor ailments: “INDICA+PRO” program]. Farmacéuticos Comunitarios. 2019;11:21-31.
